# Social Atmospherics, Affective Response, and Behavioral Intention Associated With Esports Events

**DOI:** 10.3389/fpsyg.2020.01671

**Published:** 2020-07-29

**Authors:** Wooyoung W. Jang, Kyungyeol A. Kim, Kevin K. Byon

**Affiliations:** Indiana University, Bloomington, IN, United States

**Keywords:** esports, social atmospherics, esports events, cosplay, cheering behavior

## Abstract

The purpose of this study was to conceptualize social atmospherics in the context of esports attendance and examine the relationship among social atmospherics, affective responses, and behavioral intention. Based on review literature, we conceptualized social atmospherics as five dimensions in esports events’ environments: social density, suitable behavior, similarity, cosplay, and cheering behavior. Notably, cosplay (i.e., a portmanteau of the words “costumes” and “play”) and cheering behavior factors adopted from extant social atmospherics served to capture the unique features associated with esports events. Via an online survey, data were collected (*n* = 372) from esports fans who have experienced attending esports events. The data set was split into half; the first data set (*n* = 189) was used to examine the psychometric properties of the measurement model and the second data set (*n* = 184) was employed to test the hypothesized model. The initial model fit was not shown to be acceptable. The model was re-estimated using the second data set after dropping four items with low factor loadings, resulting in the acceptable model fit. The results via structural equation modeling indicated that cheering behavior, similarity, cosplay, and social density positively and significantly influenced affective responses and behavioral intention. However, there was no significant relationship between suitable behavior and affective responses. In terms of theoretical contributions, we tested a five-factor model of social atmospherics associated with esports events and its effects on behavioral intention through affective responses. The findings in this study extend the sportscape model (Wakefield and Sloan, 1995) by incorporating the mediating effect of affective responses and expand the utility of the Stimulus–Organism–Response (SOR) framework as a viable theory that can explain esports consumption behavior.

## Introduction

Global esports revenues reached $138.7 billion in 2018, and 2.5 billion gamers across the world were expected to spend $152.1 billion in 2019 (Wilson, 2019). Such growing popularity of the esports industry has attracted interest from researchers, ranging from those interested in conceptual discussions to those seeking to conduct empirical examinations. More specifically, scholars have prompted and largely facilitated in-depth discussions regarding the definition of esports and the qualification of esports as sport and sport management scholarship (Cunningham et al., 2018; Hallmann and Giel, 2018). Researchers have also empirically examined esports consumer behavior in different contexts such as esports gameplay (Seo, 2016; Jang and Byon, 2020), online esports media consumption (Sjöblom and Hamari, 2017; Qian et al., 2019), and esports event attendance (Pizzo et al., 2018).

Despite the academic progress that has facilitated a greater understanding of esports consumers, one area that has not been explored has to do with consumers’ perceptions of social environment in esports venues. In the research regarding environment associated with traditional sport stadiums, physical environment (known as sportscape) has been viewed as important and has thus garnered considerable attention (e.g., Jang et al., 2020). More specifically, it was revealed that customers’ positive perceptions regarding physical environment led to positive emotions (Uhrich and Benkenstein, 2012; Jang et al., 2020), positive service quality perceptions (Hightower et al., 2002), and future behavioral intention (Chen et al., 2013). However, recently, researchers have emphasized the need to focus on social atmospherics (e.g., Uhrich and Benkenstein, 2012; Kim et al., 2019). Direct or indirect interactions among spectators affect their consumption experiences (Kim et al., 2019, 2020). For example, Kim et al. (2020) found that other spectators’ passionate behaviors positively influenced individuals’ perceived value, while other consumers’ dysfunctional behaviors negatively influenced individuals’ perceived value. Although research on sport service environments has seen progress, little attention has been given specifically to the esports context, especially how social atmospherics should be understood and how social atmospherics affects consumers’ affective responses and behavioral outcomes. Specifically, little is known about what elements comprise social atmospherics associated with esports events and how to understand consumers’ affective responses and behavioral intention that may result from social atmospherics in esports venue. While previous studies have demonstrated the importance of social atmospherics in managing spectators’ experiences, the sport marketing literature lacks a conceptual model that can be used to assess individuals’ perceptions of social atmospherics during esports events.

The purpose of the current study is to fill the gap in the literature by (a) conceptualizing social atmospherics in the context of esports attendance and (b) examining the interrelationships that exist among social atmospheric factors associated with esports events, consumers’ affective responses, and consumers’ behavioral intention. We define social atmospherics associated with esports events as a social aspect of the environment where service encounters take place within esports venues. The hypothesized model was proposed to explain the impact of social atmospherics associated with esports events on esports fans’ affective responses and behavioral intention. We adopted Stimulus–Organism–Response (SOR) (Mehrabian and Russell, 1974) as the theoretical foundation in the current study. Grounded in the SOR framework, we conceptualized social atmospherics in the context of esports attendance as the stimulus (S). The social atmospherics includes social density, suitable behavior, similarity, cosplay, and cheering behavior as focal constructs. In terms of delimitation, this current study intentionally adopted the selected social atmospherics factors according to the features of esports context rather than adopting all extant social atmospherics factors. For instance, physical appearance, which refers to other spectators’ appearance, has been considered as one of the important social atmospherics factors (Brocato et al., 2012). However, we did not adopt it because, unlike the traditional sport, esports spectators are likely to be less inclined yet to wear their favorite teams’ jerseys. We developed our conceptual model, including affective responses (O) and behavioral intention (R), based on the review of the literature. Then, research methods and results were presented, and we discussed the findings.

## Literature Review

### The SOR Framework

Since Mehrabian and Russell’s (1974) introduction of the SOR framework, it has been considered one of the dominant theoretical foundations for study regarding servicescape and sportscape (e.g., Kim et al., 2019; Jang et al., 2020). The SOR framework suggests a full mediation role of the organism (O) on the relationship between the stimulus (S) and the response (R) (Mehrabian and Russell, 1974). Recently, Avan et al. (2019) proposed a servicescape model based on SOR that included servicescape components and hotel guests’ emotional states and behavioral responses. In sport management literature, Jang et al. (2020) suggested a sportscape model that included sportscape factors, positive emotion, and behavioral intention across the big four United States-based major sport leagues [i.e., National Basketball Association (NBA), National Football League (NFL), Major League Baseball (MLB), and National Hockey League (NHL)]. As such, this current study employed SOR as the theoretical foundation of the research model, which includes social atmospherics associated with esports events (i.e., stimulus), affective responses (i.e., organism), and behavioral intention (i.e., response).

### Social Atmospherics of Esports Events

As previously noted, we define social atmospherics as esports consumers’ perceptions of environmental stimuli about other spectators who are simultaneously present in the esports event with a focal consumer. Although social atmospherics have been defined and used in previous studies, the uniqueness of the current study is to theorize the social atmospherics in the context of esports attendance. In the retail service context, Brocato et al. (2012) developed the other customer perception scale to measure the impact of consumer-to-consumer interactions on consumers’ behavioral intention. In addition, Uhrich and Benkenstein (2012) indicated a need for attention to be given to social stimuli in the sport service environment. As such, social elements need to be considered when consumers’ perceptions regarding the sport service environment are examined (Kim et al., 2019). There has been an emphasis especially on consumer-to-consumer interactions and the role they play in consumers’ affective responses (e.g., excitement) (Kim et al., 2019). Thus, in the present study, upon reviewing the literature, we conceptualized social atmospherics in the context of esports attendance so that we could measure esports fans’ perceptions regarding esports venue environments. The factors for social atmospherics (i.e., social density, suitable behavior, similarity, cosplay, and cheering behavior) were identified based on the review of previous peer-reviewed and press articles regarding the context of esports.

#### Social Density

Social density can be defined as an individual’s perception regarding space between him- or herself and other individuals (Uhrich and Benkenstein, 2012). Thus, in this study, we define social density as an esports spectators’ perception regarding space between spectators themselves and other spectators in an esports event environment. Mehrabian and Russell (1974) indicated that social density could significantly influence consumers’ internal and external responses. In spectator sport, “the *adequate manning* of a sporting event is reached when a high number of other customers are present in the stadium” (Uhrich and Benkenstein, 2012, p. 1744). This is because spectators in sports events have a more active role so that they can serve for social atmospherics as coperformers.

According to Jenny et al. (2018), the capacity of esports event venues is generally smaller than that of traditional sports event facilities. This might be because esports spectators must watch the entire games via an electronic screen found within esports events spaces. However, esports venue attendance has grown substantially over the last few years, which might bring venue managers’ attention to social density (i.e., perception of high numbers of other spectators) as it applies to hosting esports events. We hypothesized that social density would positively influence affective responses:

Hypothesis 1: Social density has a positive effect on affective responses.

#### Suitable Behavior

The definition of suitable behavior is an individual’s feeling or perception regarding other customers’ appropriate behaviors in the service environment (Brocato et al., 2012). Understanding suitable behavior is important because other spectators’ behaviors can influence individuals’ evaluations of their game-day experience. In the spectator sport literature, Kim et al. (2020) found that when other spectators violated codes of conduct, it resulted in negative perceptions because people have an expected social norm for others’ behavior. For example, in the context of traditional sports such as the NFL, fan brawls have been considered as one of the severe problems regarding consumption experience at sport venues (Barker, 2016). Incidents such as fighting among spectators can be an example of the violation on codes of conduct, and such unsuitable behavior is likely to lead to negative affective responses (Kim and Byon, in press). On the other hand, suitable behavior might positively influence individuals’ affective responses. According to Orland (2017), esports events are full of enthusiasm in which the crowd itself becomes a participant in the competition. In the enthusiastic esports event environment, attendees may consider other spectators’ appropriate behaviors as one of the primary social atmospherics (Kim et al., 2019). Thus, the following hypothesis was proposed.

Hypothesis 2: Suitable behavior has a positive effect on affective responses.

#### Similarity

Similarity is defined as “the extent to which an individual customer felt that they were similar to and could identify with other customers in the service environment” (Brocato et al., 2012, p. 386). When spectators are around other spectators with whom they feel similarities, they may feel more comfortable. Similarity could relate to any characteristic such as age, gender, or gamer identity. Gamer identity refers to gamer self-identification (Deshbandhu, 2016). Deshbandhu stated, “There are many players of video games but only a select few of them can be called gamers” (p. 50). Thus, gamers may be defined as an exclusive social group. Social identity theory has focused on the dimension of similarity because individuals want to be members of or included in social groups, and “the intergroup categorization leads to favoring the *in-group* and discriminating against the *out-group*” (Tajfel, 1981, p. 386). Since most esports fans have experienced esports gameplay (Jang and Byon, 2020) and because esports events attendance might signify that attendees are enthusiastic esports fans, it may be reasonable to suggest that esports event spectators are identified as gamers. Furthermore, Brocato et al. (2012) found that similarity significantly influenced consumers’ responses. Thus, we expected that the dimension of similarity would positively influence esports spectators’ affective responses.

Hypothesis 3: Similarity has a positive effect on affective responses.

#### Cosplay

Cosplay is a portmanteau word of the terms *costume* and *play*. According to Tan (2019), cosplay represents a unique culture associated with esports events. Esports fans like to cosplay their favorite in-game characters at an event venue. While cosplay-related studies have focused on people who engage themselves in cosplay activities (e.g., Rahman et al., 2012), we define cosplay as spectators’ perceptions about other spectators or professional cosplay teams’ cosplay-based actions. Notably, this concept of cosplay is similar to physical appearance, which is an important social atmospherics factor. Brocato et al. (2012) defined physical appearance as “the physical characteristics and overall look (i.e., the attributes) of other customers in the service environment (i.e., the object) as perceived by individual customers (i.e., the rater)” (p. 386). According to the theory of affordances (Gibson, 1979), consumers evaluate a service organization based on social cues such as the appearance of other consumers.

Therefore, we adopted and modified the concept of physical appearance for the cosplay construct. A consumer’s positive perception regarding other consumers’ physical appearances can lead to positive behavioral intention (Brocato et al., 2012). Thus, spectators’ perceptions regarding cosplayers’ physical appearances may play an important role in increasing spectators’ affective responses within the esports context.

Hypothesis 4: Cosplay has a positive effect on affective responses.

#### Cheering Behavior

Orland (2017) and Zacny (2015) referred to cheering behaviors as emotional shouting, booing, and using boomsticks (i.e., inflatable thundersticks) in esports events. In the current study, we adopted cheering behavior to reflect the uniqueness of esports spectators’ cheering behaviors in an esports event context. Cheering behavior (e.g., shouting, clapping, or booing) influences other spectators’ affective responses (Uhrich and Benkenstein, 2012). Chen et al. (2013) conceptualized the concept of consumer behavior to measure spectators’ passion, which is defined as spectators’ emotionally expressive behaviors resulting from strong positive arousal during sport consumption (Kim et al., 2019). Chen et al. (2013) developed a scale to measure spectators’ perceptions regarding sport stadium atmosphere, and they found that the component of stadium atmosphere positively and indirectly influenced spectators’ internal responses (i.e., satisfaction) and behavioral intention. Thus, esports consumers’ cheering behavior may significantly influence their affective response. The following hypothesis was built:

Hypothesis 5: Cheering behavior has a positive effect on affective responses.

### Behavioral Intention

In the present study, behavioral intention is defined as esports attendees’ intention to attend their favorite esports events in the future. According to SOR (Mehrabian and Russell, 1974), internal responses, such as affective responses, lead to approach or avoidance behavior regarding a given environment. Approach behavior that is the product of positive emotional responses can manifest in various ways such as on-site spending, revisiting to the event, or positive word of mouth. For instance, Uhrich and Benkenstein (2012) found a significant interrelationship that exists between affective responses and visit frequency. Chen et al. (2013) revealed the significant impact of satisfaction on behavioral intention of purchasing tickets for sports event attendance. Avan et al. (2019) adopted the intention of approach and avoidance behaviors that emerged from the influence of individuals’ emotional states. Recently, Jang et al. (2020) revealed the significant impact of positive emotion on behavioral intention regarding future attendance. Drawing upon previous studies, we investigated the impact of spectators’ affective responses on their behavioral intention. The findings in the literature led to the development of the following hypothesis:

Hypothesis 6: Affective responses have a positive effect on behavioral intention.

### Mediating Role of Affective Responses

Employing Mehrabian and Russell’s (1974) SOR framework, Bitner (1992) suggested that servicescape factors influence affective responses, which in turn leads to approach or avoidance behavior. In other words, affective responses serve as a full mediation between service environment stimuli and consumers’ behavioral intention. The direct effects of environmental cues on affective responses and the direct impact between affective states and behavioral intention have been granted attention in studies regarding the servicescape and the sportscape. Walsh et al. (2011) revealed the mediating effects of pleasure and arousal in the relationship between consumers’ cognitions regarding the store environment and customers’ behaviors. Nusairat et al. (2017) indicated the mediating role of emotion and cognition in the relationship between social cues and customer behavior in a shopping mall environment. Avan et al. (2019) found that positive and negative emotions served as mediators between servicescape components and behavioral responses. In sport management, using the SOR framework, Jang et al. (2020) revealed the mediating effect of emotion in the relationship between sportscape cues and behavioral intention in the context of professional sport leagues. As such, we postulate that affective responses mediate the relationship between social atmospherics cues associated with esports events and behavioral intention:

Hypothesis 7: Affective responses fully mediate the relationship between social atmospherics components (i.e., social density, suitable behavior, similarity, cosplay, and cheering behavior) and behavioral intention.

## Materials and Methods

### Participants and Data Collection Procedure

Data were collected from United States -based adults via Amazon Mechanical Turk (M-Turk). We recruited respondents who had a reliable record (i.e., >99% approval rating and over 100 approvals) to participate in our survey. We required that participants must have experience in attending at least one professional esports event. Thus, we asked participants about esports game titles, esports events, and the locations of the esports events to ensure that participants had experience attending esports events.

While we initially collected 400 responses, 28 were removed because of too-short completion times (e.g., <1 min). We assumed that too-short completion times were not reliable due to the lack of engagement with the survey. A total of 372 usable data remained. The demographics of the usable data were as follows. Male participants totaled 271 (72.7%), and female participants totaled 102 (27.3%). Regarding age, there were 83 (22.3%) respondents who were between 19 and 25 years of age, 208 (55.7%) who were between 26 and 35 years of age, and 59 (15.8%) who were between 36 and 45 years of age. The demographics of this sample represent the characteristics of esports consumers as a whole (Newzoo Esports, 2018). With regard to annual income, 173 (46.4%) earned incomes between $40,000 and $79,999, and 122 (32.7%) earned incomes between $10,000 and $39,999. Concerning ethnicity, Caucasians totaled 268 (71.8%), African Americans totaled 53 (14.2%), and Asians totaled 32 (8.6%).

We randomly split the data into two sets. The first data set (*n* = 189) was used to examine the psychometric properties of the measurement model, and the second data set (*n* = 184) was used to examine the hypothesized model. In terms of the demographics of the first data, male participants were 144 (76.2%), and female participants were 45 (23.8%). Forty-nine (26%) were between 19 and 25 years, 106 (56%) were between 26 and 35 years, and 34 (18%) were over 36 years. One hundred thirty-eight (73%) were Caucasians, 29 (15%) were African Americans, and Asians totaled 14 (7.4%). For the second data, male participants were 127 (69%), and female participants were 57 (31%). Thirty-four (18.5%) were between 19 and 25 years, 102 (55.5%) were between 26 and 35 years, and 48 (26%) were over 36 years. Caucasians totaled 130 (70.7%), 24 (13%) were African Americans, and Asians were 18 (9.8%).

### Instruments

The survey items related to the social atmospherics of esports events, affective responses, and behavioral intention were adapted from Wakefield and Sloan (1995) (i.e., social density), Brocato et al. (2012) (i.e., suitable behavior and similarity), Uhrich and Benkenstein (2012) (i.e., affective response), and Jang and Byon (2020) (i.e., behavioral intention). Based on the review of the literature, we adapted cosplay (three items) and cheering behavior (four items). We used items for the two constructs by modifying items of physical appearance (Brocato et al., 2012) for cosplay and perceived customer behavior (Uhrich and Benkenstein, 2012; Zacny, 2015; Orland, 2017) for cheering behavior.

Specifically, for cosplay, one of the items regarding physical appearance (i.e., “I liked the appearance of the other spectators”) was modified so that it read, “I like to see the cosplay of the other spectators.” Another item (i.e., “The other spectators looked nice”) was modified to read, “The other spectators’ cosplay looked nice.” Other items of the cosplay construct were as follows: “The other spectators were dressed as esports game characters” and “Other spectators’ cosplay looked like my type of exhibition.”

For cheering behavior, one of the items regarding perceived consumer behavior (i.e., “Fans frequently perform set maneuvers”) was modified to read, “Fans frequently performed to get a wave going through the stands and use boomsticks (stick balloons for cheering).” Another item (i.e., “There is always a great reaction to goals”) was modified to read, “There is always a great reaction to the esports teams’ performances on the big screens.” Other items of cheering behavior were as follows: “The fans shouted out a cheer of their esports teams/players names” and “The fans swooned in crescendo with each good performance and cried out in pain with every close miss” (Table 1).

**TABLE 1:** Indicator loadings (λ), construct reliability (CR), and average variance extracted (AVE) for the measurement model (the first data set, *n* = 189).

Factors and variables	λ	CR	AVE
Social density		0.78	0.55
1. The fans’ stands are confined.	0.79		
2. The fans’ stands are crowded.	0.58		
3. The fans’ stands are cramped.	0.83		
Suitable behavior		0.63	0.31
1. The behavior of the other spectators was appropriate for the setting.	0.65		
2. The other spectators were friendly toward me.	0.63		
3. I found that the other spectators behaved well.	0.60		
4. The other spectators’ behavior was pleasant.	(0.28)		
Similarity		0.76	0.39
1. I could identify with the other spectators in the facility.	0.78		
2. I am similar to the other spectators in the facility.	0.55		
3. The other spectators are like me.	0.70		
4. The other spectators come from a similar background to myself.	0.65		
5. I fit right in with the other spectators.	(0.38)		
Cosplay		0.63	0.45
1. I like to see cosplay of the other spectators.	(0.05)		
2. The other spectators were dressed in costumes of the esports game characters appropriately.	0.81		
3. The cosplay of other spectators looked like my type of exhibition.	0.83		
Cheering behavior		0.64	0.36
1. The fans shouted out a cheer of their esports teams/players names.	0.79		
2. Fans frequently perform get a wave going through the stands and use boomsticks (stick balloons for cheering).	(0.06)		
3. The fans swooned in crescendo with each good performance and cried out in pain with every close miss.	0.58		
4. There is always a great reaction to the esports team’s performances on the big screens.	0.71		
Affective responses		0.85	0.48
1. In the venue, there are amazing vibes.	0.72		
2. In the venue, you experience really strong emotions.	0.69		
3. In the venue, the atmosphere gives you goose bumps.	0.64		
4. In the venue, there’s a real thrill in the air.	0.74		
5. In the venue, you get caught up in the general euphoria	0.69		
6. In the venue, you get a real high.	0.68		
Behavioral intention		0.83	0.62
1. I plan to continue attending my favorite esports game’s events frequently.	0.78		
2. I intend to attend my favorite esports game’s events soon.	0.80		
3. I expect to continue attending my favorite esports game’s events in the near future.	0.78		

*Parentheses = the dropped items.*

Therefore, we adapted 28 items representing seven factors (i.e., social density = 3 items, suitable behavior = 4 items, similarity = 5 items, cosplay = 4 items, cheering behavior = 4 items, affective responses = 6 items, and behavioral intention = 3 items).

### Data Analysis

**FIGURE 1:**
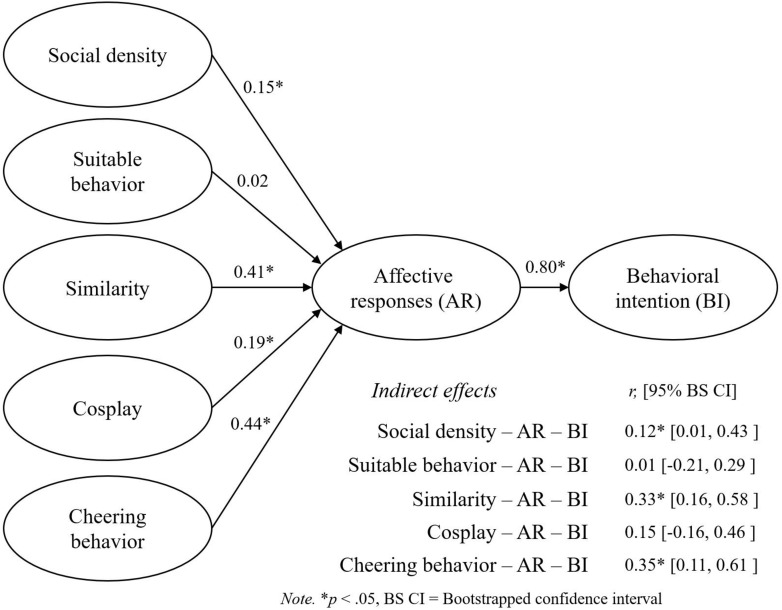
Summary of the results (the second data set, *n* = 184).

SPSS Statistics 21 was used for descriptive statistics. Using AMOS 21 with a maximum likelihood estimation method, we employed a confirmatory factor analysis (CFA) and structural equation modeling (SEM) to test the factor structure of the proposed model and the hypotheses. We validated the measurement model and the latent constructs by using CFA. Then, we used SEM to examine the links between constructs (see Figure 1).

## Results

As an assumption test for CFA, we examined the normality, outliers, and multicollinearity issues with the first data set (*n* = 184). The results of skewness (−1.05 to 0.66) and kurtosis (−0.81 to 1.39) showed the normality of the data (Hair et al., 2010). The results of the correlations for all constructs met the suggested criteria (<0.85) (Kline, 2015). The results of the variance inflation factor (1.13–4.64) showed that there were no multicollinearity issues. Lastly, the boxplot indicated that there were no outliers.

As a result of CFA, the model fit was not acceptable [χ^2^ = 1,211.61, *df* = 329, χ^2^/*df* = 3.68, Comparative Fit index (CFI) = 0.68, and root mean squared error of approximation (RMSEA) = 0.119, 90% CI = 0.13–0.20] based on the suggested cutoff values (Hair et al., 2010) of the goodness-of-fit (e.g., normed chi-square < 3.0, CFI > 0.90, RMSEA < 0.08). We found that there were four items whose factor loading values were below the threshold of 0.50 (Hair et al., 2010). These items had to do with one item of the suitable behavior factor, which read, “The other spectators’ behaviors were pleasant;” one item of the similarity factor, which read, “I fit right in with the other spectators;” one item of the cosplay factor, which read, “I like to see other spectators’ cosplay;” and one of the cheering behavior factor, which read, “Fans frequently get a wave going through the stands and use boomsticks.” These low factor loadings indicated that the items could not significantly represent the respective factors. Empirically, the low factor loading also negatively influenced the reliability and validity of the factors (Hair et al., 2010). Therefore, we decided to drop the four items.

Since we dropped the four items, the second data set (*n* = 184) was used to conduct another CFA to examine the respecified model because an independent sample should be used to validate the respecified model (Hair et al., 2010). The results showed that the model fit of the revised measurement model was good (χ^2^ = 618.31, *df* = 231, χ^2^/*df* = 2.68, CFI = 0.84, and RMSEA = 0.09, 90% CI = 0.08–0.10). Table 2 indicates that the constructs in the measurement model had acceptable convergent validity because the factor loadings were above the suggested threshold of 0.50 (Hair et al., 2010). The average variance extracted (AVE) also suggested adequate convergent validity of the measurement model because the values were 0.50 or higher. For discriminant validity, we used the squared correlation between factors and AVE (Table 3). Overall, the squared correlations were smaller than the AVE values (Fornell and Larcker, 1981), which indicates that the constructs in the measurement model have discriminant validity, so items represent well its construct that the items belong. Additionally, all of the composite reliability (CR) values were above the suggested criteria (>0.07), indicating good reliability of the constructs in the measurement model. Thus, the results of CFA showed that the measurement model was found to be good reliability and validity.

**TABLE 2:** Indicator loadings (λ), construct reliability (CR), average variance extracted (AVE) for the measurement model (the second data set, *n* = 184).

Factors and variables	λ	CR	AVE
Social density		0.75	0.50
1. The fans’ stands are confined.	0.82		
2. The fans’ stands are crowded.	0.51		
3. The fans’ stands are cramped.	0.76		
Suitable behavior		0.75	0.50
1. The behavior of the other spectators was appropriate for the setting.	0.74		
2. The other spectators were friendly toward me.	0.72		
3. I found that the other spectators behaved well.	0.66		
Similarity		0.82	0.54
1. I could identify with the other spectators in the facility.	0.80		
2. I am similar to the other spectators in the facility.	0.71		
3. The other spectators are like me.	0.75		
4. The other spectators come from a similar background to myself.	0.66		
Cosplay		0.75	0.60
1. The other spectators were dressed in costumes of the esports game characters appropriately.	0.74		
2. The cosplay of other spectators looked like my type of exhibition.	0.81		
Cheering behavior		0.77	0.53
1. The fans shouted out a cheer of their esports teams/players names.	0.77		
2. The fans swooned in crescendo with each good performance and cried out in pain with every close miss.	0.64		
3. There is always a great reaction to the esports team’s performances on the big screens.	0.77		
Affective responses		0.88	0.56
1. In the venue, there are amazing vibes.	0.82		
2. In the venue, you experience really strong emotions.	0.80		
3. In the venue, the atmosphere gives you goose bumps.	0.80		
4. In the venue, there’s a real thrill in the air.	0.74		
5. In the venue, you get caught up in the general euphoria.	0.68		
6. In the venue, you get a real high.	0.64		
Behavioral intention		0.88	0.70
1. I plan to continue attending my favorite esports game’s events frequently.	0.82		
2. I intend to attend my favorite esports game’s events soon.	0.82		
3. I expect to continue attending my favorite esports game’s events in the near future.	0.87		

**TABLE 3:** Interfactor correlation (the second data set, *n* = 184).

	AVE	SD	SB	SM	CP	CB	AR	BI
SD	0.50	1						
SB	0.50	0.13* (0.02)	1					
SM	0.54	0.13* (0.02)	0.62* (0.38)	1				
CP	0.60	0.33* (0.11)	0.64* (0.41)	0.61* (0.37)	1			
CB	0.53	0.19* (0.04)	0.61* (0.37)	0.55* (0.30)	0.59* (0.35)	1		
AR	0.56	0.67* (0.45)	0.67* (0.45)	0.75* (0.56)	0.75* (0.56)	0.75* (0.56)	1	
BI	0.70	0.57* (0.33)	0.57* (0.33)	0.82* (0.67)	0.61* (0.37)	0.80* (0.64)	0.53* (0.28)	1

*p < 0.05. Parentheses = squared correlation. SD = social density; SB = suitable behavior; SM = similarity; CP = cosplay; CB = cheering behavior; AR = affective responses; BI = behavioral intention.

Then, we examined SEM with the second data set (*n* = 184) to examine the hypotheses. The structural model showed a reasonable fit to the data (χ^2^ = 654.24, *df* = 236, χ^2^/*df* = 2.77, CFI = 0.83, and RMSEA = 0.09, 90% CI = 0.08–0.10). The results of SEM (Table 4) showed that the following four latent variables positively and significantly influenced affective responses: social density (β = 0.15, *p* < 0.05), similarity (β = 0.41, *p* < 0.05), cosplay (β = 0.19, *p* < 0.05), and cheering behavior (β = 0.44, *p* < 0.05). These results supported hypotheses 1, 3, 4, and 5. However, there was a nonsignificant relationship between suitable behavior and affective responses (β = 0.02, *p* > 0.05), and thus, hypothesis 2 was not supported. Affective responses positively and significantly affected behavioral intention (β = 0.80, *p* < 0.05), supporting hypothesis 6. The explanatory power (*R*^2^) of affective responses (i.e., 88%) and behavioral intention (i.e., 65%) also support the precise prediction of esports spectators’ emotional and behavioral responses.

**TABLE 4:** Results of structural equation modeling (the second data set, *n* = 184).

	β	*t*-Value	Hypothesis
**Direct effects**			
Social density – affective responses	0.15*	2.69	H1: Supported
Suitable behavior – affective responses	0.02	0.20	H2: Not supported
Similarity – affective responses	0.41*	5.18	H3: Supported
Cosplay – affective responses	0.19*	2.05	H4: Supported
Cheering behavior – affective responses	0.44*	5.30	H5: Supported
Affective responses – behavioral intention	0.80*	9.90	H6: Supported
**Indirect effects**	*r*, (95% BS CI)		
Social density – behavioral intention	0.12* (0.01, 0.43)		
Suitable behavior – behavioral intention	0.01 (−0.21, 0.29)		
Similarity – behavioral intention	0.33* (0.16, 0.58)		
Cosplay – behavioral intention	0.15 (−0.16, 0.46)		
Cheering behavior – behavioral intention	0.35* (0.11, 0.61)		H7: Partially supported

*p < 0.05. BS CI, bootstrapped confidence interval.

We used a bootstrapping procedure to examine the mediation effects of affective responses. Specifically, we employed 95% confidence intervals using 2,000 bootstrap samples. There were three significant and indirect relationships between social density [β = 0.12, *p* < 0.05, 95% BC CI (0.01, 0.43)], similarity [β = 0.33, *p* < 0.05, 95% BC CI (0.16, 0.58)], cheering behavior [β = 0.35, *p* < 0.05, 95% BC CI (0.11, 0.61)], and behavioral intention through affective responses. However, suitable behavior [β = 0.01, *p* > 0.05, 95% BC CI (−0.21, 0.29)] and cosplay [β = 0.15, *p* < 0.05, 95% BC CI (−0.16, 0.46)] were found to be insignificant. The results indicated that there were mediating effects of affective responses in the relationships of three social atmospherics with the behavioral intention, which partially supported hypothesis 7. Although the two hypotheses about suitable behavior (i.e., the hypotheses 2 and 7) were not supported, the full set of the identified five factors of social atmospherics should be considered essential in the context of esports attendance. We discuss the results in the following section.

## Discussion

The findings in this current study extend current knowledge in that they describe esports event participants’ perceptions regarding the social atmospherics of the esports event environment. These perceptions can explain esports event participants’ emotional and behavioral responses. While there is interest in and an understanding regarding the importance of social atmospherics in sport marketing, servicescape, and sportscape, sport management researchers’ understanding of social atmospherics of esports events’ environments has been limited. We proposed the conceptualization of social atmospherics regarding esports events’ environments, including the characteristics of esports events (i.e., cosplay and cheering behavior), to provide theoretical support for the social atmospherics in the context of esports events. According to the findings, the suggested conceptual model in this current study was found to be reliable, and it predicted esports spectators’ emotional and behavioral responses.

Specifically, cheering behavior, similarity, cosplay, and social density were revealed as significant social atmospherics components that could lead to positive affective responses and behavioral intention. According to social identity theory (Tajfel, 1981), the intergroup categorization, such as in-group feelings, is important for the generation of similarity perceptions among consumers, which is important because similarity leads consumers to have positive affective responses. Additionally, the findings provide empirical evidence of the concepts of cosplay and cheering behavior, which are unique features of the esports event environment. While pressed articles exist that discuss the phenomena associated with esports events, such as cosplay (Tan, 2019) and cheering activities (Zacny, 2015; Orland, 2017), there were limited peer-reviewed articles. Previous studies regarding cosplay have been born of an interest in participants engaging cosplay activities (e.g., Rahman et al., 2012). Based on the concepts of customer-to-customer and physical appearance (Brocato et al., 2012), the present study conceptualized cosplay as individual spectators’ perceptions regarding other spectators or professional teams’ cosplay activities. Cheering behavior was also modified from the concept of customer behavior (Uhrich and Benkenstein, 2012) to assess it within the context of esports events. Future studies may need to improve the social atmospherics associated with esports events by modifying the cosplay or cheering behavior factors or adding new factors. Lastly, social density significantly influenced affective response and behavioral intention. This finding supports the line of research regarding social atmospherics in sports venues (Uhrich and Benkenstein, 2012; Kim et al., 2019). In other words, perceptions of a high number of other spectators have a positive impact on spectators’ affective responses in esports arena.

However, there was no significant impact of suitable behavior on affective responses, which was unexpected. Unsuitable behavior, such as dysfunctional behavior (Kim et al., 2020), is found to be problematic in traditional sport event contexts, so the unexpected finding in the current study might have to do with the differences between esports fans and traditional sports fans. For instance, Nelson (2019) stated that esports fans’ way of supporting their favorite teams and players are more similar to following a lifestyle brand than a sport team. Therefore, the code of conduct regarding suitable behaviors of other spectators in esports event contexts might be different from the spectators’ code of conduct in traditional sport event contexts. Although suitable behavior was not found to significantly impact affective responses or behavioral intention in this current study, we still believe that suitable behavior needs to be adopted and examined again in future studies after it is reconceptualized and modified per the features of the esports context.

### Theoretical Contributions

According to the overview of the findings, we indicate two primary theoretical contributions. First, we conceptualize social atmospherics in the context of esports attendance. Drawing upon the literature, social atmospherics were adapted in the esports spectating environment. Notably, the items and constructs regarding cosplay and cheering behavior were revealed as the unique features of the esports attendance environment. Furthermore, similarity and social density were also shown as an important social atmospherics at esports venues. As stated above, suitable behavior needs further validation in future studies. By extending social atmospherics in a new context, esports attendance, our findings contributed to the line of research regarding social atmospherics in sports venues (Uhrich and Benkenstein, 2012; Chen et al., 2013; Kim et al., 2019) and retail settings regarding servicescape (Brocato et al., 2012; Nusairat et al., 2017; Avan et al., 2019).

Second, grounded in the SOR framework (Mehrabian and Russell, 1974), the current study proposed a conceptual model that includes social atmospherics components to predict esports spectators’ behavioral intention through their affective responses. Despite there being two extant studies regarding esports event attendance (Pizzo et al., 2018) and esports venue (Jenny et al., 2018), there was no study related to esports event participants’ perceptions of social atmospherics in an esports venue. The current study theoretically contributes to the line of research regarding esports fans’ consumption behaviors by suggesting a conceptual model aimed at assessing social elements in the esports events environment and predicting esports event participants’ affective responses and behavioral intention. In addition, the results indicated that affective responses acted as a means of mediation between social atmospherics cues and behavioral intention, which supports the SOR framework. The previous studies in servicescape (e.g., Walsh et al., 2011; Nusairat et al., 2017; Avan et al., 2019) and sportscape (e.g., Uhrich and Benkenstein, 2012; Jang et al., 2020; Kim et al., 2019) have determined the generalizability of the SOR framework, and the current study contributes to the extension of the SOR framework in terms of the social environment in esports event contexts.

### Practical Contributions

The results indicated the satisfactory reliability and validity of the constructs in the model and adequately explained the variability of esports event participants’ affective responses and behavioral intention. As important triggers for positive affective responses, the results suggested cheering behavior, similarity, cosplay, and social density. When esports event organizers or managers develop their marketing strategies, they need to consider the impact of social atmospherics factors. We found that cheering behavior is the strongest trigger for positive affective responses. For instance, Orland (2017) stated that cheering experience at esports events might lead esports fans to attend on-site events rather than to watch events via the various media platforms; watching esports games on on-site electronic screens is technically the same as attending on-site events in person.

Thus, in order to manage esports event attendees’ experiences effectively, managers may need to prepare cheering promotions by distributing items such as free esports teams’ jerseys or boomsticks (i.e., a pair of long balloons for cheering) in order to promote an intergroup feeling. Furthermore, the promotions, such as holding cosplay contests among spectators or inviting professional cosplay teams to an event, may positively influence esports event attendees’ affective responses and behavioral intention. For instance, Sneaky, former Cloud9 professional player in League of Legends league, has cosplayed numerous in-game champions, and it has caught a lot of esports fans’ attention. Lastly, esports managers may need to attract more esports events attendees in the future because the size of esports arenas has been growing so that it might be difficult to fill in the esports arena for social density. In 2020, the largest public esports arena on the West Coast is being built in Los Angeles (Gonzalez, 2020). The size will be 26,000 ft^2^, which is equivalent to 5.5 NBA basketball courts. The esports events in this public esports arena need to prepare strategies to fill in the large arena with spectators, and manage the crowd. While the perception of high numbers of other customers can lead to positive affective responses from esports attendees, managers may need to effectively manage safety issues on the days of the esports events.

Finally, the findings showed the impact of social atmospherics in esports attendance context on their affective responses and future behavior. As such, if esports organizers can successfully manage the perception regarding the suggested social atmospherics, it may positively influence esports attendees’ emotions and revisit intention. In this sense, esports event organizers or managers need to consider social atmospherics to improve their managing and marketing activities.

### Limitations and Suggestions for Future Studies

Although the findings of the current research contribute to the extant and emerging literature on esports event environments, there are limitations related to further validation of the items. Despite the conceptual model showing good reliability and validity, in the first attempt of CFA, four items were removed for empirical reasons (i.e., lower factor loadings). We should acknowledge that two items represented a revised cosplay construct (i.e., the construct without one cosplay item initially proposed), even though three or more items preferably (Hair et al., 2010). While the relationship between a revised cosplay construct and affective responses was statistically significant (*p* = 0.04), it was marginally less than the threshold of 0.05. It might explain the insignificant indirect effects of the relationship between cosplay and behavioral intention. This can be addressed by improving the items in future studies. Future studies may need to use more adequate words to improve the items. If the items are modified with more proper wording, the revised items might better represent their constructs so that the latent variables can be measure more adequately.

In addition, future studies may need to consider extending the conceptual model proposed by the current study by adding moderators such as a ticket price. Although one of the implications of this study is that it serves to fill a gap regarding the need to explore social atmospherics in esports events contexts, esports attendees’ game-day experience might be heterogeneous per their stands’ section. For example, according to the online ticketing website, the ticket prices for the esports event of Overwatch at the Met Philadelphia (i.e., the Philadelphia Metropolitan Opera House) range from $49 to $232 per section. Since esports venue attendance has grown substantially over the last few years (Jenny et al., 2018), future studies may need to pay more attention the ticket price as a moderator because esports attendees’ heterogeneous game-day experience based on their stands section (i.e., ticket price) might influence on the relationship between social atmospherics and their affective responses.

## Data Availability Statement

The raw data supporting the conclusions of this article will be made available by the authors, without undue reservation.

## Ethics Statement

The studies involving human participants were reviewed and approved by the Institutional Review Board at Indiana University – Bloomington. Written informed consent for participation was not required for this study in accordance with the national legislation and the institutional requirements.

## Author Contributions

WJ contributed by serving as the primary writer of the manuscript and by performing the primary data collection and data analysis. KK contributed by providing critical feedback, assistance in the data analysis, and suggestions to the initial and revised drafts. KB provided insight into conceiving the overall research idea, design, and execution. He contributed immensely to the overall improvement in the quality of this study by giving critical feedback based on his profound insight into the research. All authors contributed to the article and approved the submitted version.

## Conflict of Interest

The authors declare that the research was conducted in the absence of any commercial or financial relationships that could be construed as a potential conflict of interest. The reviewer JZ declared a past collaboration with one of the authors KB to the handling Editor.
